# Effect of hCG application at different moments of the estrous cycle on corpus luteum and uterine vascularization and serum progesterone concentration in mares

**DOI:** 10.21451/1984-3143-AR2018-0103

**Published:** 2019-10-24

**Authors:** Maria Augusta Alonso, Luciano Andrade Silva, Fernanda Jordão Affonso, Kleber Menegon Lemes, Eneiva Carla Carvalho Celeghini, Renata Lançoni, Henrique Fulanetti Carvalho, Rubens Paes de Arruda

**Affiliations:** 1 Laboratory of Semen Biotechnology and Andrology, Department of Animal Reproduction, School of Veterinary Medicine and Animal Science, University of Sao Paulo (USP), Pirassununga, São Paulo, Brazil.; 2 Laboratory of Theriogenology Dr. O.J. Ginther, Department of Veterinary Medicine, School of Animal Sciences and Food Engineering, University of Sao Paulo, Pirassununga, São Paulo, Brazil.

**Keywords:** Color Doppler ultrasonography, Equine, Horse, Embryo transfer

## Abstract

Establishment of pregnancy after embryo transfer is the ultimate goal of an embryo transfer program and increasing pregnancy rates and reducing pregnancy loss are mandatory. The utilization of treatments to improve conception rates in recipient mares has been the focus of several research groups over the last years and the results are controversial. Some studies using human chorionic gonadotrophin (hCG) found promising results. Our hypothesis was that hCG administration would cause an additional stimulation on luteal function, uterine and luteal vascularization and progesterone concentration, and the mares would have increased uterine and cervix tone. Therefore, in the present study the effects of hCG administration to induce ovulation, on day 0 (day of ovulation) or day 5 postovulation were evaluated on corpus luteum characteristics, reproductive tract vascularization, and serum progesterone concentration from ovulation until day 15 postovulation. Groups were: G1: (control) - no hCG; G2: 2500 IU of hCG to induce ovulation when a follicle greater than 35mm and uterine edema were detected; G3: 2500 IU hCG on day 0; G4: 2500 IU hCG on day 5 postovulation. Twelve mares were randomly assigned to each group, during consecutive cycles, in a Latin Square experimental design, in a total of 48 cycles. Doppler ultrasound evaluations were performed daily from day 0 until day 15 postovulation, including mesometrial vascularity, endometrial vascularity and corpus luteum vascularity. Blood samples were collected for serum progesterone concentration. Data was analyzed using the Proc Glimmix SAS Procedure for nonparametric variables and Proc Mixed for parametric parameters. There was no treatment effect for all variables studied (P > 0.05). Characteristics were only affected by day (P < 0.05). It can be concluded that hCG administration at the time points suggested in the current study did not alter the characteristics evaluated.

## Introduction

Conventional embryo transfer in mare has been continuously growing over the last years, and the most recent numbers show a total of 29,651embryo flushes and 21,321 transfered embryos worldwide in 2015. Also, advanced reproductive technologies such as Intracytoplasmatic Sperm Injection (ICSI) and cloning are gaining visibility and being more frequently used each year to produce embryos to be transferred. Therefore, stablishment of pregnancy after embryotransfer is the ultimate goal of an embryo transfer program and increasing pregnancy rates and reducing pregnancy loss are mandatory.

The study of treatments in order to improve conception rates in recipient mares has been the focus of several research groups over the last years and the results are controversial. Commercially, embryo transfer centers use their own protocol in order to achieve good results, such as nonsteroidal anti-inflammatory drugs, GnRH analogs, hCG and progestin/progesterone supplementation. Our group has focused on the use of hCG, based on results of increased pregnancy rates and reduction of pregnancy loss found in cattle and sheep (Nephew at al. 1994; Schmitt *et al*., 1996; Saharrea *et al*., 1998; Santos *et al*., 2001; Khan *et al*., 2003; Beindorff *et al*., 2009; Vecchio *et al*., 2010). Our previous studies aimed to improve pregnancy rates and increase the percentage of adequate recipient mares on the day of transfer. A greater percentage of mares that received hCG on the day of ovulation or day 1 postovulation showed adequate uterine and cervical characteristics, such as echogenicity and tone compared to control mares. Moreover, recipient mares that received hCG had a tendency to have higher pregnancy rates than untreated, buserelin or norgestomet treated mares (Fleury *et al*., 2007). Kohne *et al*., (2014) found an increase in maternal serum circulating progestins along with an increase of the embryo proper, suggesting an improvement of uterine environment, whereas others have detected a negative effect, showing a decrease on pregnancy rate when hCG was used (McCue *et al*., 2012).

Interestingly, hCG receptors in reproductive tract in other species have been reported, suggesting indirect effects, other than directly in the ovary and CL. However, it had been shown that hCG has direct effects on uterine vascularization in swine (Ziecik *et al*., 1986), rat (Hermesteiner *et al*., 1999) and human (Jauniaux *et al*., 1992).

No studies were performed comparing effects of three different timings of hCG administration during the estrous cycle on uterine and ovarian vascularity and corpus luteum morphology and progesterone concentration from the day of ovulation until day 15 postovulation in the same group of mares. Our hypothesis was that hCG administration would cause an additional stimulation on luteal function, uterine and luteal vascularization and progesterone concentration, and the mares would have increased uterine and cervix tone. Therefore, in the present study the effects of hCG administration to induce ovulation, on day 0 (day of ovulation) or day 5 postovulation were evaluated on corpus luteum characteristics, reproductive tract vascularization, and serum progesterone concentration from ovulation until day 15 postovulation.

## Materials and Methods

### Animals

The experiment was conducted between the months of December of 2011 and April 2012. Twelve Mangalarga and mixed-Mangalarga mares, between 5 and 15 years of age (Mean 10.75) were used after a thorough clinical reproductive examination during which no alterations were detected. All mares were cycling normally and had at least two consecutive cycles before entering the experiment. Mares were in body condition score of 5 or greater (Henneke *et al*., 1983). Animals were housed in pasture with Coast Cross grass (*Cynodon dactyolon* (L) Pers), mineral salt and water *ad libitum*. Immediately before ultrasound examination, animals received 1 kg of a 13% crude protein concentrate ration (Guabi^®^). This study was approved by the Ethic Committee (2265/2011) in the use of animals of the School of Veterinary Medicine and Animal Science of University of São Paulo, respecting all the Ethical Principles in Animal Research. Animals were housed at Fazenda Santa Rita II, Piracaia, Sao Paulo (Latitude 23^o^ 03’14”W; longitude 46^o^ 21’29”), Brazil.

### Study Design

All mares were submitted to treatment groups randomly, using an 4x12 latin square. An operator blind to the treatment type conducted all palpations and ultrasound examinations. Treatment groups were: Group 1 (control mares) did not receive any treatment; Group 2 (hCG induction) 2500IU of hCG were administered (Vetecor, Hertape Calier^®^) intravenously when a 35 mm follicle and uterine edema were detected; Group 3 (hCG day 0) 2500IU of hCG were administered intravenously the day of ovulation; Group 4 (hCG day 5) 2500IU of hCG were administered intravenously on day 5 postovulation. The days of hCG administration were chosen according to the following criteria: Group 1 served as contro, Group 2 received treatment as is recommended by the manufacturer, Group 3 received treatment on a day we hypothesized would increase progesterone concentrations and Group 4 received treatment because we hypothesized it would improve the CL function.

On day 16 postovulation, all mares received 5 mg of intramuscular dinoprost tromethamine (Lutalyse^®^; Pharmacia). Twelve cycles per treatment, with a total of 48 cycles were analyzed.

### Reproductive Tract Examination

Mares were examined every other day until a follicle of 30 mm was detected. Then, they were examined daily by transrectal palpation and ultrasound scanning until ovulation was confirmed. Examinations were performed from the day of ovulation until day 15 postovulation between 0700 and 1100. All evaluations were performed by the same examiner. Uterine tone was evaluated daily from day 0 until day 15 postovulation through transrectal palpation using digital compression as described by Hayes and Ginther (1986). Scoring varied from 1 to 4, 1 refering to a flacid uterus and 4 a firm uterus. Cervical tone was examined by compressing the cervix over the rectum floor (Ginther, 1992a) and score 1 was used to describe a flaccid cervix and 4 a tight cervix.

### Ultrasonography

A M5VET ™ (Mindray Medical International Limited, China) equipped with a linear probe (6 - 8 MHz) and Color Doppler was used for all the examinations. Ultrasound examinations were performed daily once a 30 mm follicle was detected. In group 2, when a 35 mm follicle with uterine edema was detected, hCG was administered. Follicle growth was measured daily until ovulation.

Once the corpus luteum was formed, two perpendicular diameters were measured in a frozen image and an average was calculated. Corpus luteum area (cm^2^) was determined using the software provided within the ultrasound. Both measurements were performed daily from day 0 until day 15 postovulation.

**Figure 1:**
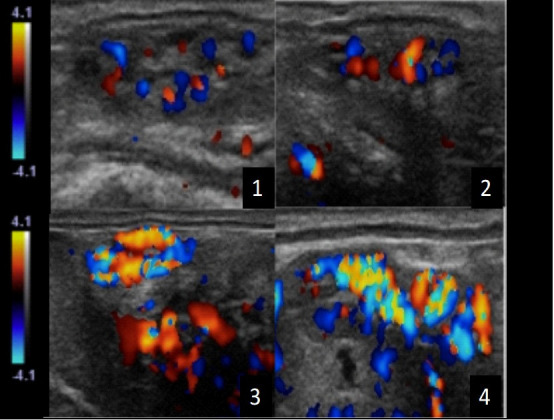
Color-Doppler sonograms representing scores used to evaluate ovarian pedicle vascularization in mares. The scores indicated minimal (1) to maximal (4) vascularity.

**Figure 2:**
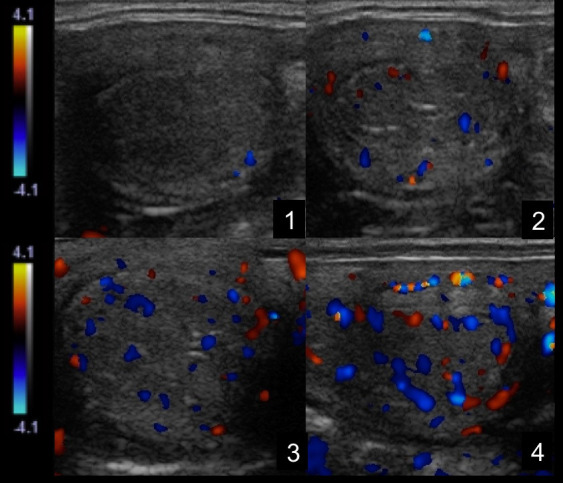
Color-Doppler sonograms representing scores used to evaluate endometrium vascularization in mares. The scores indicated minimal (A) to maximal (D) vascularity.

All ultrasonographic evaluations were performed using the same ultrasound and Doppler settings were maintained throughout all examinations and executed daily from day 0 until day 15 postovulation. Corpus luteum vascularity was evaluated using Color Doppler and a grade from 0 to 100% vascularization was attributed after a one-minute scan of the entire structure, as previously described (Ginther *et al*., 2007). Ovarian pedicle (Fig. 1), endometrium (Fig. 2) and mesometrial attachment were subjectively scored (score 1 was minimun and 4 maximun vascularity) (Ginther *et al*., 2007). Using spectral Doppler mode, resistance index of the ovarian pedicle ipsilateral to the corpus luteum and bilateral mesometrial attachment was calculated using the package available in the ultrasound machine after three similar waveforms were obtained using spectral mode (Ginther and Utt, 2004; Silva *et al*., 2005; Ferreira *et al*., 2008). In order to detect differences in uterine vascularization between ipsi and contralateral uterine horns, a statistical analysis was perfomed. When no difference was detected, the average between the two horns was considered.

### Progesterone concentration

Blood samples were collected daily from day 0 until day 15 postovulation imeddiately before the ultrasound examinations. Blood was centrifuged, and serum was stored at -20^o^C for further analysis of serum progesterone concentration. Analysis was perfomed using the radioimunnoassay Coat a Count, Siemens at the Laboratorio de Dosagens Hormonais - LDH at the School of Veterinary Medicine and Animal Science of the University of Sao Paulo. For the progesterone assay interassay variation was 2.97% and intra-assay variation 5.9% and ther minimal detectable concentration was 0.003 ng/mL.

### Statistical Analysis

Parametric data (CL diameter and area and serum progesterone concentration) were evaluated with the Kolmogorov-Smirnov test for normality. When data was not normally distributed it was transformed to natural logarithms.

Comparison among treated and control groups were analysed for effects of group and day and interaction of group with day. SAS mixed procedure (9.2version; SAS Institute, Inc., Cary, NC, USA) was used. Non-parametric data (vascularity indexes, echogenicity, uterine and cervical tone) were evaluated using Glimmix procedure for SAS. When effect of day was detected, diferences were examined using the paired t student test. Statistical difference was considered significant when P < 0.05. Data are presented as Mean ± Standard Error of the Mean.

## Results

In our experiment, the first evaluation performed was the comparison of serum progesterone concentration between mares that had a single versus double ovulations, regardless of their groups. This comparison showed a different progesterone concentration profile, where double ovulators had a decrease of progesterone concentration two days after the single ovulators, therefore we decided to exclude the animals with double ovulations. Five cycles presented double ovulations, three of which were from the same animal and the two remaining from different individuals. Double ovulations occured on days 1, 2, 3, 6 and 8 after the first ovulation. Thus, the above excluded cycles were not considered for the analysis.

**Figure 3:**
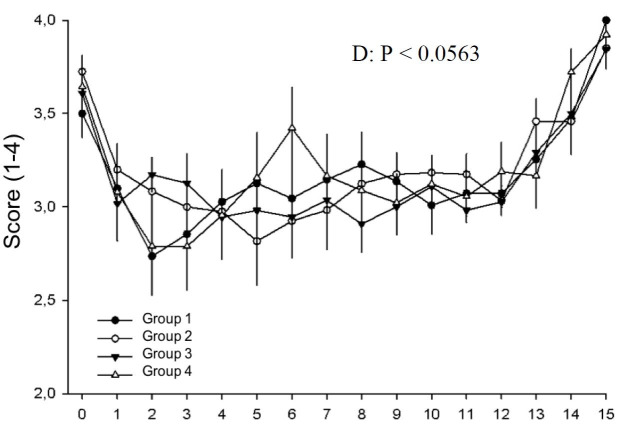
Uterine morphoecogenicity in different treatment groups during days 0 to 15 postovulation in mares. *indicates statistical difference.

Uterine morphoecogenicity was not influenced by treatments, however it tended to be influenced by day. Uterus was heterogenous, presenting edema on day 0. From days 1 to 12 postovulation a more homogenous appearance was detected, without detectable edema. From day 13 up to day 15 edema was again detected (Fig. 3).

**Figure 4:**
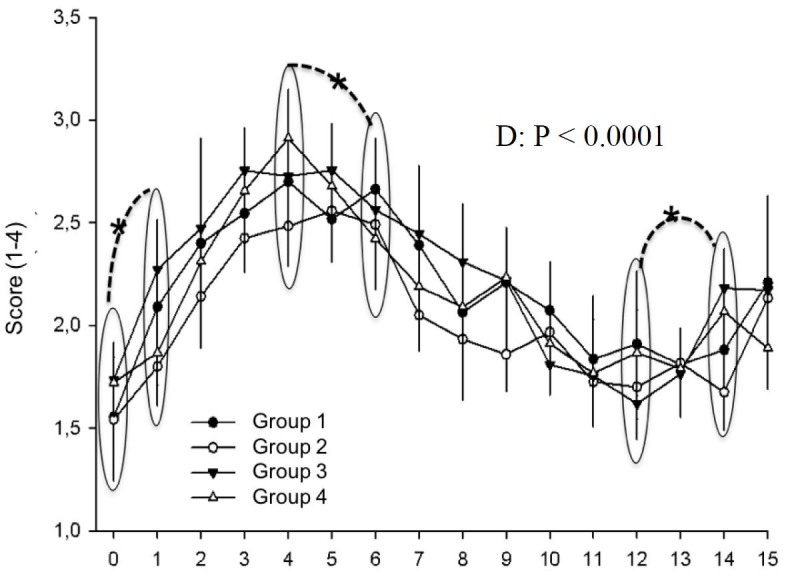
Uterine tone in different treatment groups during days 0 to 15 postovulation in mares. *indicates statistical difference.

Uterine tone was affected by day (P < 0.05) and not treatment (P > 0.05). On the day of ovulation, uterus was flaccid. A statistica significant (P < 0.05) decrease was detected between days 0 and 1 postovulation, and maintained until day 4, when it reached its higher score. Between days 4 and 6 a statisticaly significant decrease was detected. The decreasing pattern persisted until day 12. However, between days 12 and 14 an increase was found and maintained until day 15 (Fig. 4).

**Figure 5:**
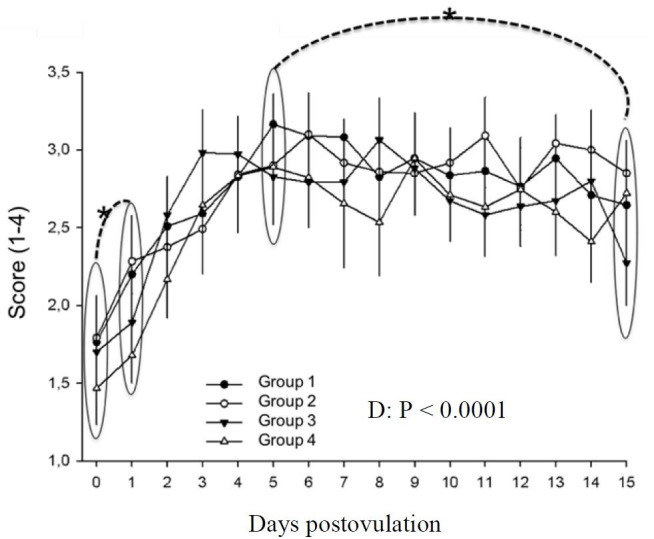
Cervical tone in different treatment groups during days 0 to 15 postovulation in mares. *indicates statistical difference.

Cervical tone was not affected by treatments. Day, however, affected this characteristic (P < 0.0001). On day 0, the tone was minimal and increased significantly on day 1 (P < 0.05). The increase hapenned until day 5 and did not change until day 15 (P < 0.05; Fig. 5).

The corpus luteum area was affected by day (P < 0.0001), and not by treatment. Area increased from day 0 to day 1 (P < 0.0001) and between days 3 and 4 a decrease was noted (P <0.05) and continued until day 15 postovulation (Fig. 6). The same was described for corpus luteum diameter, with no treatment effect. Day significantly affected corpus luteum diameter (P < 0.0001). An increase between days 0 and 1 was found (P < 0.0001) and a decrease is initiated between days 3 and 5 (P < 0.0004), persisting until day 15 postovulation (Fig. 7).

**Figure 6:**
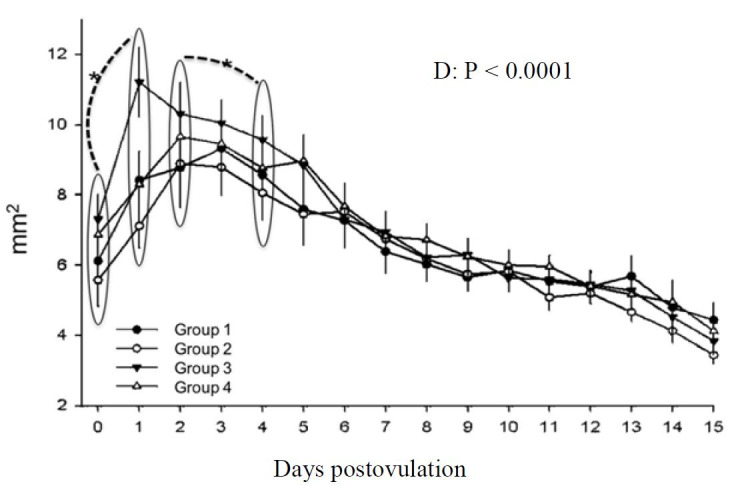
Corpus luteum area in mares in different treatment groups during days 0 to 15 postovulation in mares. *indicates statistical difference.

**Figure 6:**
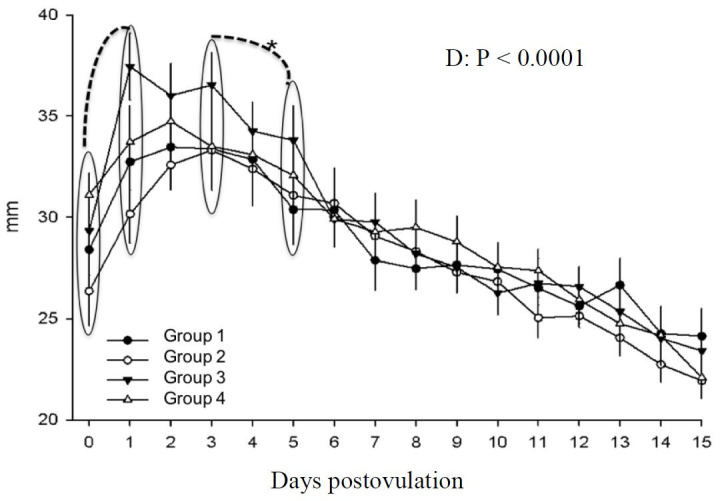
Corpus luteum area in mares in different treatment groups during days 0 to 15 postovulation in mares. *indicates statistical difference.

**Figure 8:**
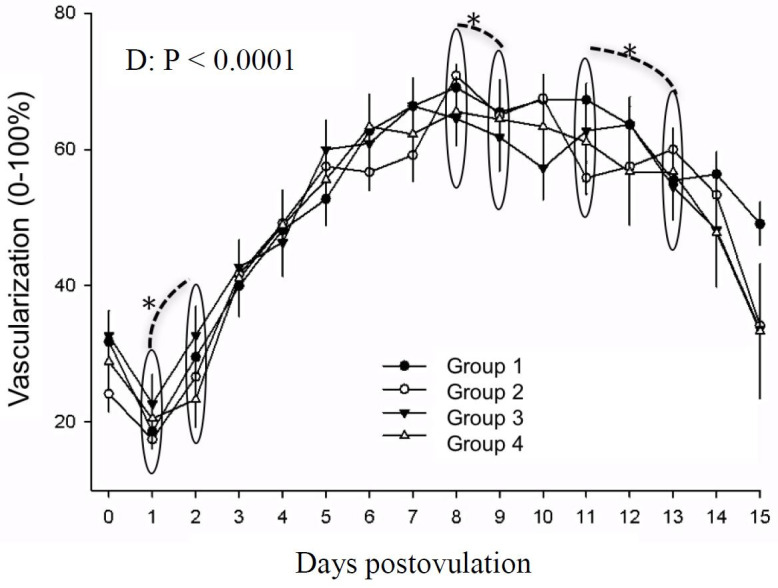
Corpus luteum vascularization (0-100%) in mares in different treatment groups during days 0 to 15 postovulation in mares. *indicates statistical difference.

Corpus luteum vascularization was also affected by day (P < 0.0001). On day 0, vascularization was low and increased statistically between days 1 and 2 (P < 0.05) and maintained until day 8. Between days 8 and 9 a decrease was noted (P < 0.05) until day 11. Another decrease was seen between day 11 and 13 (P < 0.05), which continued until day 15 postovulation (Fig. 8).

**Figure 9:**
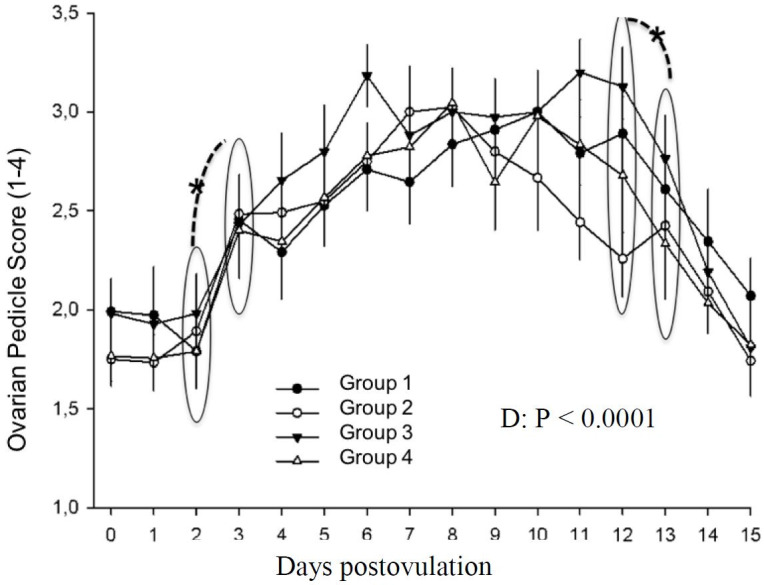
Ovarian pedicle score in mares in different treatment groups during days 0 to 15 postovulation in mares. *indicates statistical difference.

Ovarian pedicle vascularization was affected only by day (P < 0.0001). Low values were found on the day of ovulation until day 2. A significant increase occured between day 2 and 3 (P < 0.0001), reaching maximun value on day 6 and was constant until day 12. A decrease was found between days 12 and 13, continuing until day 15 (Fig. 9).

**Figure 10:**
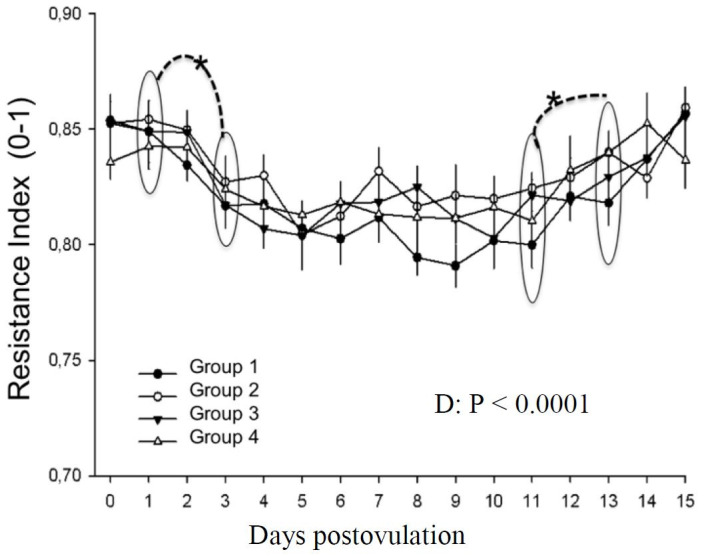
Resistance index of the ovarian pedicle in mares in different treatment groups during days 0 to 15 postovulation in mares. *indicates statistical difference.

Likewise, resistance index of the ovarian pedicle presented a day effect (P < 0.0001). On days 0 and 1, values were higher. A significant decrease occured between days 1 and 3 (P < 0.0001) and maintained until day 11 when a significant increase was detected, continuing until day 15. Treatment effect was not found (P > 0.05; Fig. 10).

Mesometrial attachment resistance index did show day effect (P < 0.0001). Higher resistance index was found on days 0 and 1, and a decrease significantly occured between days 1 and 3 (P < 0.0001), remaining low until day 5. Bewteen days 5 and 9, an increase was noted (P < 0.05), continuing until day 13. Again, between days 13 and 15, values decreased significantly (P < 0.05).

**Figure 11:**
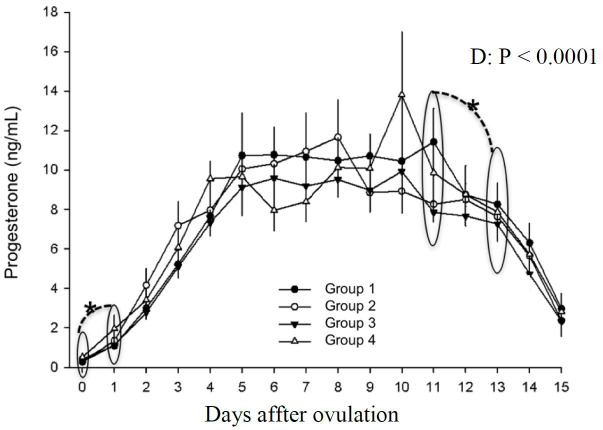
Progesterone concentration in mares in different treatment groups during days 0 to 15 postovulation in mares. *indicates statistical difference.

Treatment did not alter serum progesterone concentrations but was influenced by the day (P < 0.0001). Comparing days 0 and 1, a significant increase occured (P < 0.0001), until day 5 postovulation. Levels remained constant between days 5 and 11. Between days 12 and 14 a decrease was detected (P < 0.05) and persisted until day 15 postovulation (Fig. 11).

## Discussion

High pregnancy rates after embryo transfer are mandatory for the success of embryo transfer programs. Therefore, several studies have focused on methods of increasing these rates. A wide variety of treatments are utilized in the field despite controversial results regarding their efficacy and usefulness, such as non-steroidal anti-inflamatory medications (Kobliscke *et al*., 2008; Kobliscke *et al*., 2010; Hartman, 2011); antibiotic (Hartman, 2011); hCG (Fleury *et al*., 2007; Hartman, 2011; McCue *et al*., 2012), deslorelin (McCue *et al*., 2012) and progestogens (DeLuca *et al*., 2011). This study is the first study reporting hCG utilization in three different times of the estrous cycle in mares, for inducing ovulation, on the day of ovulation and on day 5 postovulation and its effect on reproductive tract vascularity, corpus luteum, cervical and uterine tone and serum progesterone concentration throughout 15 days after ovulation.

It is well known that good uterine and cervical tone on the day of embryo transfer results in better pregnancy rates (Carnevale *et al*., 2000). Furthermore, based on our previous results (Fleury *et al*., 2007), the percentage of mares to which hCG was administered on day 1 that presented adequate characteristics, good uterine and cervical tone and homogenous uterus on day 5, was greater compared to control mares. However, in our present findings hCG injected in all the three times did not alter uterine and cervical tone (Figures 4 and 5). The increased uterine tone observed in this study from day 0 to 1 post-ovulation, was referred to the increased progesterone levels which dissipates endometrial edema (Pelehach *et al*., 2002). Change to whereas maximum uterine tone observed on day 4 post-ovulation contradict with Bonafos *et al*. (1994) who recorded a peak on day 6 postovulation. Between days 4 and 6 postovulation, the first significant decrease was noted, reaching minimun values on days 12 and 13. This finding is also different from the minimun values on day 10 obtained by Bonafos *et al*. (1994). Interestingly, an increase was detected between days 12 and 14, which continued until day 15. A possible explanation is the increase in estrogen concentrations associated with low progesterone concentration but still above 2ng/ml, which resembles the early pregnancy physiology where a maximun uterine tone was found (Hayes and Ginther, 1986). Treatments did not change cervical tone during the period evaluated, disagreeing with our previous study that found an increase in the percentage of recipient mares on day 6 postovulation with good cervical tone when mares received hCG on day 1 postovulation (Fleury *et al*., 2007). The controversy of the presented results might be due to the experimental design, the current study evaluated the same mares in all four treatments while the previous used different mares in each group (Fleury *et al*., 2007). According to the current results, no effect on reproductive tract tone was noted. However, cervical tone was flaccid on the day of ovulation, in agreement with the literature (Hayes and Ginther, 1986). Between days 0 and 1, a significant increase was detected, probably in response to the augment in progesterone level. The maximum tone was observed on day 5 and remained with diestrus tone (closed) until day 15 postovulation, when a decline occured, in parallel with progesterone decrease.

Corpus luteum diameter and area (Figures 67) were measured and no hCG effect was noted, as stated by Kohne *et al*. (2014) who treated mares to induce ovulation or on day 5 with hCG and found no changes in CL dimensions. In bovine, it is suggested that hCG has a direct effect on the CL, changing its cellular component percentages, size and progesterone production (Hansel and Blair, 1996). However, in the current study only day affected the characteristics evaluated, in agreement with (Fleury *et al*., 2007). As described in the literature, after ovulation an increase in CL area occurs and the maximum area and diameter are found on day 2, remains high until day 4 and a gradual decrease until day 15 postovulation occurs (Bergfelt and Adams, 2007). Our results show an increase in area from day 0 to day 1, when the CL is initially formed. On days 2 and 3, maximum area was found. A decrease from day 2 to day 4 occurred and continued progressively until day 15. This measured decrease parallels progesterone increases as previously described by Arruda *et al*. (2001).

We hypothesized that hCG could affect CL vascularization and perfusion based on studies that detected changes in uterine blood flow in other species such as swine (Ziecik *et al*., 1986) and rats (Rao and Alsip, 2007) and also has an effect on angiogenesis (Tsampalas *et al*., 2010). In the present study, no effect of treatment was detected. Corpus luteum vascularization increased from day 0 until day 8, reaching its maximum value when maximum progesterone concentration values also occured. Our results are in agreement with Bollwein *et al*. (2002) who found maximum vascularization on day 6 and Ginther *et al*. (2007) who reported maximum values between days 6 and 8 postovulation. In all treatments, between days 8 and 9 a decrease on luteal vascularization was noted, antecipating a decrease on progesterone concentration (Figure 8), similar to data reported by Ishak *et al*., (2017). The vascularization values decreased until day 15, associated with the decline in progesterone concentration. This decrease of vascularization during the preluteolytic (day 14 postovulation) and luteolytic periods (15 to 17 days postovulation) was previously described by Ginther *et al*. (2007), compatible with its corresponding physiological moments.

Ovarian pedicule vascularization correlated with corpus luteum vascularization but was not altered by any treatment (Figure 9). Probably, endogenous LH levels were adequate to form a normal vascularized CL, therefore an additive effect of LH receptor stimulation does not cause an alteration on CL formation when hCG is given to induce ovulation or on the day of ovulation. Additionally, when given on day 5 postovulation, CL vascularization was already stablished, and no alterations were noted.

Endometrial and mesometrial vascularization were also measured and both uterine horns had the same pattern, in agreement with Silva *et al*. (2005). hCG did not affect uterine vascularization, differing from reports found in rats showing a direct and dose dependent effect of hCG injection and vasodilation and reduced resistance index in uterine and mesenteric arteries (Hermsteiner *et al*., 1999). Hendriks *et al*. (2006) used hCG on day 7 postovulation in mares and were also unable to detect an effect of treatment on uterine vascularization, in accordance with Biermann *et al*. (2014). Endometrial vascularization increased from day 1 to 2 postovulation, reaching maximum values on days 3 and 5, in agreement with Ignácio *et al*. (2012) and Bollwein *et al*. (1998). One possible explanation is the emergence of the first follicular wave that occurs during diestrus, increasing estrogen leves (Silva *et al*., 2005). It can also be discussed that during the beginning of diestrus, an increase in blood flow allows the effect of progesterone on its uterine receptors, causing the increase in uterine tone and endometrial gland secretions preparing for a pregnancy. Between days 5 and 6, a decline in endometrial vascularity perfusion occurs until day 10. From day 10 to 13 a new increase occurs until day 15, as shown in the literature (Ginther, 2007).

Our data did not show any progesterone alterations due to treatments. Profiles were the same for treated and control mares, differening from previous studies which detected progesterone increase after hCG treatments on the day of embryo transfer in recipient mares (Fleury *et al*., 2007) or on days 3, 4 and 5 (Kelly *et al*., 1988). hCG may not be beneficial in individuals with normal corpus luteum formation and progesterone secretion on cycles during the physiological breeding season (Figure 11). Another possibility to explain a lack of response of the CL to hCG application is that receptors belonging to the G Family, one of which is the hCG/LH receptor, downregulate when a prolonged agonist stimilus happens, causing its internalization and degradation by lysosomes (Ghinea *et al*., 1992).

Also, an inadequate CL formation during the breeding season is not a common event; an appropriate supply of LH ensures a normal CL development and progesterone production. This might be the reason why supplementing with hCG does not cause an increase in progesterone concentration. On the other hand, altered luteal development has been reported associated with ovulations during transitional periods (King, 2011), periods during which the magnitude of LH surge is decreased for the first ovulation, maybe in part resulting from low estrogen levels (Ginther, 1992b) or fall transition, when LH levels are already declining (starting during the ovulatory season) (Silvia *et al*., 1986). Studies to evaluate the use of hCG during spring and autumn transition on CL formation, lifespan, function and pregnancy rates and losses would be of great value.

It can be suggested that there is a difference in hCG utilization comparing equine with bovine, caprine and ovine. The suggested effect of hCG on CL function, with secondary corpora lutea formed and increased progesterone concentrations (Nephew *et al*., 1994; Schmitt *et al*., 1996; Saharrea *et al*., 1998; Santos *et al*., 2001; Khan *et al*., 2003; Campanile *et al*., 2008; Beidorff *et al*., 2009) was not detected in mares using the protocols in the present study.

In previous studies performed by our group, a positive effect of hCG injection on the day of embryo transfer revealed higher pregnancy rate compared to the control mares (Fleury *et al*., 2007), differing from McCue *et al*. (2012) that found a negative effect of hCG when used at the same moment. In the present study, a fertility trial was not conducted, therefore a study comparing pregnacy rates would be important.

Studies on the use of hCG in horses to improve reproductive characteristics and pregnancy rates are still limited when compared to those found in other species such as bovine and ovine (Santos *et al*., 2001; Khan *et al*., 2003; Campanile *et al*., 2008; Beidorff *et al*., 2009; Rizos *et al*., 2012). Results are controversial, and several practitioners use hCG on the day of the transfer in order to improve pregnancy rate and other treatments such as progesterone supplementation and antiiflamatory drugs, despite conflicting results (Koblischke *et al*., 2008, 2010; Hartman, 2011). However, high pregnancy rates are achieved after embryo transfer with no additional treatments when a suitable recipient mare with good reproductive characteristics is used (Allen, 2001; Lopes *et al*., 2011).

In summary, in the present study hCG treatment for ovulation induction, on day of ovulation or on day 5 postovulation did not increase uterine and corpus luteum vascularization as hypothesized. CL diameter and area also were not changed among treatment groups and control. Moreover, uterine and cervical tone were not increased during the studied period on neither treatment. Finally, hCG treatments did not increase serum progesterone concentration.
